# Effects of the circulating environment of COVID-19 on platelet and neutrophil behavior

**DOI:** 10.3389/fimmu.2023.1130288

**Published:** 2023-03-14

**Authors:** Alexander T. Fields, Elizabeth A. Andraska, Christof Kaltenmeier, Zachary A. Matthay, Kimberly Herrera, Brenda Nuñez-Garcia, Chayse M. Jones, Katherine D. Wick, Silvia Liu, Jian-Hua Luo, Yan-Ping Yu, Michael A. Matthay, Carolyn M. Hendrickson, Roland J. Bainton, Tessa J. Barrett, Jeffrey S. Berger, Matthew D. Neal, Lucy Z. Kornblith

**Affiliations:** ^1^ Department of Surgery, University of California, San Francisco, Zuckerberg San Francisco General Hospital, San Francisco, CA, United States; ^2^ Trauma and Transfusion Medicine Research Center, Department of Surgery, University of Pittsburgh, Pittsburgh, PA, United States; ^3^ Division of Pulmonary, Critical Care, Allergy and Sleep Medicine, Department of Medicine, University of California, San Francisco, San Francisco, CA, United States; ^4^ Cardiovascular Research Institute, University of California, San Francisco, San Francisco, CA, United States; ^5^ Department of Pathology, School of Medicine, University of Pittsburgh, Pittsburgh, PA, United States; ^6^ Department of Anesthesia and Perioperative Care, School of Medicine, University of California, San Francisco, San Francisco, CA, United States; ^7^ Leon H. Charney Division of Cardiology, Department of Medicine, New York University (NYU) Grossman School of Medicine, New York, NY, United States; ^8^ New York University (NYU) Center for the Prevention of Cardiovascular Disease, New York University (NYU) Langone Health, New York, NY, United States; ^9^ Division of Vascular Surgery, Department of Surgery, New York University (NYU) Grossman School of Medicine, New York, NY, United States

**Keywords:** blood platelets, neutrophil extracellular traps, COVID-19, thromboinflammation, plasma

## Abstract

**Introduction:**

Thromboinflammatory complications are well described sequalae of Coronavirus Disease 2019 (COVID-19), and there is evidence of both hyperreactive platelet and inflammatory neutrophil biology that contributes to the thromoinflammatory milieu. It has been demonstrated in other thromboinflammatory diseases that the circulating environment may affect cellular behavior, but what role this environment exerts on platelets and neutrophils in COVID-19 remains unknown. We tested the hypotheses that 1) plasma from COVID-19 patients can induce a prothrombotic platelet functional phenotype, and 2) contents released from platelets (platelet releasate) from COVID-19 patients can induce a proinflammatory neutrophil phenotype.

**Methods:**

We treated platelets with COVID-19 patient and disease control plasma, and measured their aggregation response to collagen and adhesion in a microfluidic parallel plate flow chamber coated with collagen and thromboplastin. We exposed healthy neutrophils to platelet releasate from COVID-19 patients and disease controls and measured neutrophil extracellular trap formation and performed RNA sequencing.

**Results:**

We found that COVID-19 patient plasma promoted auto-aggregation, thereby reducing response to further stimulation *ex-vivo*. Neither disease condition increased the number of platelets adhered to a collagen and thromboplastin coated parallel plate flow chamber, but both markedly reduced platelet size. COVID-19 patient platelet releasate increased myeloperoxidasedeoxyribonucleic acid complexes and induced changes to neutrophil gene expression.

**Discussion:**

Together these results suggest aspects of the soluble environment circulating platelets, and that the contents released from those neutrophil behavior independent of direct cellular contact.

## 1 Introduction

Thromboinflammatory complications are well described sequalae of Coronavirus Disease 2019 (COVID-19) (1), mediated by cellular and non-cellular thrombotic and inflammatory derangements resulting from SARS-CoV-2 infection (2, 3). Platelets are known as a crucial link between thrombosis and inflammation, and in COVID-19 there is evidence of alterations to platelet behavior including to platelet hemostatic function, cytokine production (4, 5), and effector cell properties demonstrated by induction of neutrophil release of extracellular traps (6, 7) and promotion of an inflammatory hypercoagulable endotheliopathy (3).

Platelets are classically known for their role in thrombus formation and hemostasis; however, evidence of their interaction with and regulation of immune cells and participation in inflammatory processes, both infectious and non-infectious, have been a crucial link in understanding thromboinflammatory diseases (8, 9). In the setting of COVID-19 platelets have been found to have altered functional responses and gene expression profiles (4, 10), as well as induce myeloid and endothelial cell activation (3, 4, 11). As is the case in many inflammatory and infectious diseases, a variety of inflammatory and thrombosis-related signaling molecules are released from platelets and other cell types in COVID-19 patients, including serotonin, platelet factor 4, and monocyte chemoattractant protein 1 (5, 7, 12–14). Increased platelet activity and associated release of cytokines recruit leukocytes, the most abundant being the neutrophil, which can engage in a number of pro-inflammatory immune behaviors contributing to COVID-19 symptoms and outcomes. Biologically, these pro-inflammatory behaviors include formation of neutrophil extracellular traps (NETs), in a process referred to as NETosis (15, 16). NETosis is a link in the thromboinflammatory continuum, as NETs have been observed in microvascular occlusions in COVID-19 patient lung tissue that was also co-enriched in platelet factor 4 (7). Together these observations suggest a high degree of crosstalk between thrombotic and inflammatory systems, and evidence that this crosstalk may not require direct cell-cell interactions, but that the soluble microenvironment may also play a key role. In fact, in other diseases characterized by thromboinflammation, there is also evidence that the circulating soluble environment can alter platelet behavior (17–19). However, what role the circulating soluble environment of COVID-19 has on platelet and neutrophils remains incompletely elucidated. Here we test the hypotheses that 1) plasma from COVID-19 patients can induce a prothrombotic platelet functional phenotype, and 2) contents released from platelets (platelet releasate) from COVID-19 patients can induce a proinflammatory neutrophil phenotype.

## 2 Methods

### 2.1 Patient samples

Patient samples were collected with the approval of the University of California, San Francisco’s Institutional Review Board (IRB). Whole blood samples were collected under an initial waiver of consent from adults (>18 years) undergoing evaluation for possible COVID-19 on arrival to the Zuckerberg San Francisco General Hospital (ZSFG) emergency department based on fever, respiratory symptoms (cough, shortness of breath, upper respiratory infection, wheezing), gastrointestinal symptoms (diarrhea, nausea/vomiting), and/or changes in taste or smell as part of the overarching COVID-19 Associated Coagulopathy, Inflammation, and Thrombosis (Co-ACIT) Study. COVID-19 diagnosis in these patients was based on a positive or negative SARS-CoV-2 polymerase chain reaction (PCR) test performed in the course of clinical care. This created tandem cohorts of COVID-19 patients and symptom-matched disease control patients. COVID-19 patients and disease controls were followed until discharge from the emergency department or hospital, and comprehensive patient characteristics, physiological and laboratory parameters, and clinical outcomes were collected.

### 2.2 Assays of patient samples

On whole blood samples from COVID-19 patients, and disease controls, clotting function was assessed by Thromboelastography (TEG) using a TEG6s analyzer (Haemonetics, Boston, MA) with the global hemostasis and lysis cartridge, which performs a resonance-based quantitative assessment of clot formation, maintenance and breakdown. Platelet aggregation was assessed pre- and post-stimulation with agonists adenosine diphosphate (ADP), collagen, a PAR1 agonist hexapeptide (thrombin-receptor activating peptide-6 (20), arachidonic acid, or ristocetin, using multiple electrode impedance aggregometry *via* the Multiplate analyzer (Roche, Basel, Switzerland), according to manufacturer instructions. Agonists were purchased from Hart Biologicals (Hartlepool, UK). Biomarkers of thrombosis and inflammation present in plasma were measured by enzyme-linked immunosorbent assays (ELISA).

### 2.3 Preparation of plasma and platelet releasate

Plasma was isolated by centrifugation at 2960xg for 10 minutes and stored at -80°C. Contents released from platelets (platelet releasate) were generated as previously described (3). Briefly, whole blood was centrifuged at 200xg for 10 minutes, and resulting platelet rich plasma was centrifuged at 1000xg for 10 minutes in the presence of 1:10 acid citrate dextrose solution A anticoagulant. The resulting platelets were resuspended in HEPES-buffered Tyrode’s solution with 1μM Prostaglandin-E1 (Cayman Chemical) and counted. The platelets were pelleted again and resuspended at 5x10^5^/μL in HEPES-buffered Tyrode’s and supplemented with 2mM CaCl_2_ to allow for content release. The resulting platelet releasate was collected and stored at -80°C. Healthy donor whole blood samples were obtained and processed in the same way for the purpose of having control treatment groups in the *ex-vivo* experiments described below.

### 2.4 *Ex-vivo* platelet assays

*Ex-vivo* platelet assays were performed based on previously described methods (17). Briefly, platelets from healthy donors were isolated by centrifugation at 200xg for 20 minutes to generate platelet rich plasma, which was then centrifuged to generate a platelet pellet, which was washed and resuspended in HEPES-buffered Tyrode’s solution supplemented with 3mg/mL bovine serum albumin. The platelet count was adjusted to 2x10^5^/μL. The platelets were allowed to rest for 30 minutes and then treated with 12% (v/v) patient plasma for 30 minutes (19). Treated platelets were subjected to measurements of adhesion and aggregation by microfluidic adhesion assays and multiple electrode impedance aggregometry, respectively. Microfluidic adhesion assays were performed as previously described (21, 22). Briefly, treated platelets were perfused through a parallel plate flow chamber (Ibidi, Grafelfing, Germany) coated with 100μg/mL of collagen (Rat tail Type I, Corning) and thromboplastin (Extem reagent, Werfen, Barcelona, Spain) at approximate venous shear (100s^-1^) for 10 minutes. In order to avoid artifactual attachment, unattached platelets were washed out of the chamber with HEPES-buffered Tyrode’s solution at approximate arterial shear (1000s^-1^) for 20 minutes (22). Attached platelets were counted and imaged under phase contrast at 60x magnification. Area of attached platelets was quantified using FIJI (23). Aggregometry was performed as described above with the agonist collagen (Hart Biologicals) to parallel the collagen coating in the adhesion assays. Data were analyzed using Excel (Microsoft) and R (www.r-project.org). For presentation, all *ex-vivo* treated platelet data were normalized to autologous healthy control plasma treatment of matched parallel samples of platelets isolated and treated on the same day (i.e., platelets treated with plasma from the same donor).

### 2.5 *Ex vivo* neutrophil assays

Neutrophils isolated from healthy donors were exposed to platelet releasate from COVID-19 patients and disease controls, and neutrophil extracellular trap (NET) formation was assessed by ELISA for myeloperoxidase (MPO)-deoxyribonucleic acid (DNA) and gene expression changes by ribonucleic acid (RNA) sequencing.

#### 2.5.1 Neutrophil isolation and stimulation

Whole blood was drawn from a single male donor (to limit donor heterogeneity for sequencing) with sodium heparin, treated with 3% dextran, and then washed at 500g for 5 min. Red blood cells were lysed with H_2_0 for 30 seconds followed by staining with CD66b PE (Thermo Fisher) and CD16 APC (Thermo Fisher). Cells were sorted using a BD FACSAria. Cells were then plated at a density of 150,000 cells per well. Wells rested for one hour and were then treated with platelet releasate (1:2 dilution) for four hours. Positive control wells were treated with PMA (250nM) or Ionomycin (2.5ug/ml). All experiments were performed in duplicates. After four hours of treatment the supernatant was removed for NETosis capture ELISA.

#### 2.5.2 NETosis quantification

NETosis was quantified with an ELISA detecting MPO-DNA complexes (24). After treatment with platelet releasate or positive control, cell supernatant was aspirated and incubated on a 96 well plate from a commercial MPO ELISA kit (Hycult biotech) and then NETs were measured using MPO (Roche) associated with DNA ELISA (Roche). Samples were analyzed on a plate reader at 405nm. Each sample was plated in duplicate, and values were reported as average of the duplicates.

#### 2.5.3 Neutrophil bulk RNA sequencing

Following four hours of treatment with platelet releasate, stimulated neutrophils were harvested and washed twice in PBS (500g x 5 min). RNA was isolated according to the TRIzol Reagent protocol (Invitrogen). RNA integrity (RIN) was determined, and samples were further processed if RIN > 7 and purity of > 1.9 (260nm/280nm). The sequencing core at the University of Pittsburgh was given at least 100ng RNA in duplicates for further sequencing. Samples were sequenced using NovaSeq6000 platform (Illumina). The sequencing reads were then quality controlled. Reads were aligned by STAR aligner. Based on aligned RNA-seq data, gene counts were quantified for each library. Differential expression analysis was performed with R package DESeq2. Differentially expressed genes (DEGs) were defined by false discovery rate (FDR)≤5%, and DEGs with changes larger than 1.5-fold were further applied into Ingenuity Pathway Analysis (Qiagen) to detect pathways enriched by the gene alterations. Significant pathways were defined by FDR≤ 5%.

**Figure 1:**
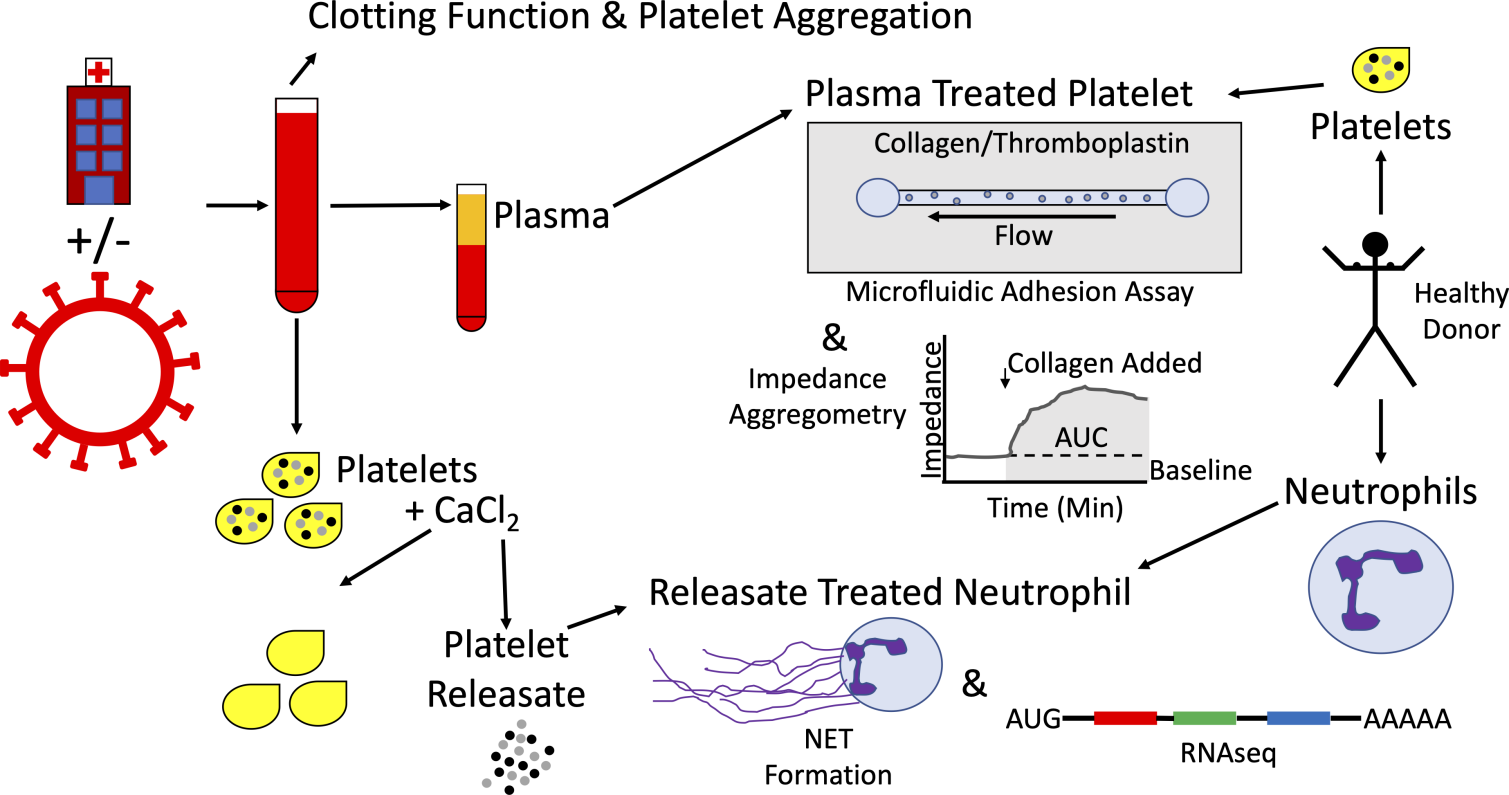
Schematic representation of experimental strategy. Patients being evaluated for COVID-19 were enrolled on arrival to the emergency department, yielding tandem cohorts of COVID-19 patients and symptom-matched disease control patients. Whole blood was collected and clotting function and platelet aggregation were assessed by thromboelastography and multiple electrode impedance aggregometry, respectively. Whole blood was processed into plasma and stored at -80°C. Platelets were isolated, exposed to calcium chloride, and the resulting platelet releasate was collected and stored at -80°C. Separately, platelets were isolated from healthy donors, exposed to plasma samples, and their adhesion and aggregation properties were studied using a microfluidic parallel plate flow chamber and multiple electrode impedance aggregometry, respectively. Neutrophils were isolated from healthy donors and exposed to platelet releasates from COVID-19 patients and disease controls. Neutrophil extracellular trap (NET) formation was assayed and neutrophil RNA was sequenced.

The above experimental strategies are represented schematically in **Figure 1**.

## 3 Results

### 3.1 Coagulation and inflammation characteristics of plasma and platelet releasate donors

#### 3.1.1 Plasma donors

The demographics, clinical characteristics, global clotting function, platelet aggregation function, and thrombosis and inflammation biomarkers of the COVID-19 patient and disease control plasma donors are shown in **Table 1**. In general, COVID-19 patient plasma donors had some evidence of a prothrombotic and proinflammatory biomarker profile relative to disease controls. This is evidenced by trends toward elevated levels of prothrombotic biomarkers such as fibrinogen (median 477.5 vs 355mg/dL), and antiplasmin (median 72 vs 65.7% activity), increased clot strength due to fibrinogen (median TEG Functional Fibrinogen maximum amplitude 35.45 vs 23.4mm), and increases in platelet aggregation in response to stimulation with ADP (median 67.5 vs 40 AUC units) and ristocetin (median 81 vs 56 AUC units) in COVID-19 patients relative to disease controls. Additionally, COVID-19 patients had a trend toward elevated levels of proinflammatory biomarkers such as Interferon γ-inducible protein 10 (IP10, median 450 vs 77.5pg/mL), interleukin 10 (IL-10, 13.6 vs 6.7pg/mL), and receptor for advanced glycation end-products (RAGE, median 3091.6 vs 720.4 pg/mL) relative to disease controls.

**Table 1:** Characteristics of plasma donors.

Characteristic		Disease controls (n=12)	COVID-19 patients (n=14)
Age (yrs)		57 (19)	60 (8)
Sex (n)	Female	6	5
	Male	6	8
Race (n)	Asian	1	3
	Black/African American	3	1
	Native American or Alaskan	1	0
	Unknown/Not reported/Other	1	2
	White	6	7
Ethnicity (n)	Not Hispanic/Latino	9	6
	Hispanic/Latino	3	7
BMI (mg/kg^2^)		21.25 (6.93)	28.85 (30.77)
Platelet Count (x10^9^/L)		276 (145)	221 (115)
Leukocyte Count (x10^9^/L)		6.45 (3.18)	8.2 (3.3)
Hemoglobin (g/dL)		13.65 (2.23)	13.8 (2.9)
Hospital Days		7 (7.75)	8 (12)
ICU Days		0 (0.5)	0 (1)
Intubated on Day 1 (n)	No	11	11
	Yes	1	2
Vital Status at Discharge (n)	Alive	12	12
	Dead	0	1
Maximum O_2_ Requirement on Day 1 (L/minute)		0 (0-15)	4 (0-40)
Ventilator Days		0 (0)	0 (0)
Admission Acuity (n)	ED only	1	0
	Floor	6	8
	Floor, then ICU	1	2
	ED to ICU	3	3
	Unknown	1	0
TEG Reaction Time (minutes)		6.5 (0.7)	6.7 (0.6)
TEG Maximum Amplitude (mm)		66.9 (6.3)	67.8 (3.7)
TEG Functional Fibrinogen Maximum Amplitude (mm)		23.4 (13.3)	35.45 (12.23)
TEG Lysis (%)		0.2 (0.6)	0.3 (0.7)
Aggregometry Baseline Impedance (Resistance Units)		1363 (106)	1400 (59)
Aggregometry ADP AUC (Units)		40 (41)	67.5 (62.3)
Aggregometry Collagen AUC (units)		72 (44.8)	101 (52.8)
Aggregometry Thrombin Analog AUC (units)		86.5 (60)	105 (26)
Aggregometry Arachidonic Acid AUC (units)		42 (40.5)	56.5 (65.75)
Aggregometry Ristocetin AUC (units)		56 (48)	81 (77)
PAI-1 (pg/mL)		2.5 (1.4)	4.5 (7.7)
Antiplasmin (% Activity)		65.7 (24.6)	72 (29)
Fibrinogen (mg/dL)		355 (124)	477.5 (190)
D-dimer (μg/dL)		1 (3)	1.2 (0.8)
Factor VIII (% Activity)		236 (288.3)	181.5 (117)
vWF (% Activity)		332 (282.6)	281 (227)
VEGF (pg/mL)		15.8 (12.3)	25.2 (17.4)
Thrombomodulin (pg/mL)		6252 (2449)	4871 (2450)
Plasminogen (% Activity)		110 (36)	120.5 (48.8)
Protein C (% Activity)		65.7 (24.6)	72 (29.12)
P-selectin (pg/mL)		27.4 (25.8)	30.4 (24.8)
tPA (pg/mL)		3081.4 (3007.1)	2342 (1621)
IL-8 (pg/mL)		13.21 (11.1)	14.5 (8.7)
TNFR1 (pg/mL)		1091.4 (575.1)	1413.7 (771.9)
Ang1 (pg/mL)		5481.1 (8740.3)	6372 (7815)
Ang2 (pg/mL)		1657.3 (1245.6)	1013.3 (805.6)
MMP8 (pg/mL)		2854.4 (3442.3)	3032.1 (4079)
SPD (pg/mL)		5286 (4140)	2723 (2965)
TREM1 (pg/mL)		215.3 (144.1)	219.8 (90.3)
IL-18 (pg/mL)		234.5 (207.1)	366.4 (146.4)
Cell-free Hgb (pg/mL)		72.8 (286.4)	41.21 (148.23)
IP10 (pg/mL)		77.5 (188.5)	450 (424.2)
IL-10 (pg/mL)		6.7 (4.1)	13.6 (12)
RAGE (pg/mL)		720.4 (840.5)	3091.6 (3230.9)

Data are reported as median (interquartile range), or in the case of maximum O_2_ requirement, median (minimum-maximum). yrs, years; BMI, body mass index; ED, emergency department; ICU, intensive care unit; TEG, thromboelastography; ADP, adenosine diphosphate; AUC, area under the curve; PAI-1, plasminogen activator inhibitor 1; vWF, von Willebrand Factor; VEGF, vascular endothelial growth factor; tPA, tissue plasminogen activator; IL-8, interleukin 8; TNFR1, tumor necrosis factor receptor 1; Ang1, angiopoietin 1; Ang2, angiopoietin 2; MMP8, matrix metalloprotease 8; SPD, surfactant protein D; TREM1, triggering receptor expressed on myeloid cells 1; IL-18, interleukin 18; Hgb, hemoglobin; IP10, interferon-gamma induced protein 10; IL-10, interleukin 10; RAGE, receptor for advanced glycation end products.

#### 3.1.2 Platelet releasate donors

The demographics, clinical characteristics, global clotting function, platelet aggregation function, and thrombosis and inflammation biomarkers of the COVID-19 patient and disease control platelet releasate donors are shown in **Table 2**. Overall, similar to the plasma donors, COVID-19 platelet releasate donors had prothrombotic and proinflammatory biomarker profiles compared to disease controls. COVID-19 patients had a trend toward elevated levels of prothrombotic biomarkers such as PAI-1 (median 4.5 vs 2.6pg/mL), plasminogen (median 101.5 vs 78% activity), and fibrinogen (median 393 vs 318mg/dL), evidence of increased clot strength (median TEG overall maximum amplitude 67.3mm vs 63.7mm, and Functional Fibrinogen maximum amplitude 26 vs 20.5mm), decreased clot breakdown (median TEG Lysis 0.1 vs 1.1%), and increased platelet aggregation in response to stimulation with ADP (median 69 vs 58 AUC units) and ristocetin (median 108 vs 59.5 AUC units). Additionally, COVID-19 patients had increases in inflammatory biomarkers such as IP10 (median 429.1 vs 22.4pg/mL), IL-10 (median 9.3 vs. 1.3pg/mL), and RAGE (median 2476 vs. 686pg/mL), when compared to disease controls.

**Table 2:** Characteristics of platelet releasate donors.

Characteristic		Disease controls (n=5)	COVID-19 patients (n=5)
Age (years)		67 (9)	48 (13)
Sex	Female	0	2
	Male	5	3
Race	Asian	1	1
	Black/African American	0	0
	Native American or Alaskan	0	0
	Unknown/Not reported/Other	0	0
	White	4	4
Ethnicity	Not Hispanic/Latino	4	1
	Hispanic/Latino	1	4
BMI		27.1 (9.3)	28.95 (6.65)
Platelet Count (x10^9^/L)		254 (31)	250 (59)
Leukocyte Count (x10^9^/L)		8 (1.5)	6.5 (1.4)
Hemoglobin (g/dL)		12.9 (0.9)	14.3 (2.9)
Hospital Days		3 (3)	2 (7)
ICU Days		0 (0)	0 (0)
Intubated on Day 1	No	5	4
	Yes	0	1
Vital Status at Discharge	Alive	5	4
	Dead	0	1
Maximum O_2_ Requirement on Day 1 (L/minute)		0 (0-3)	1 (0-40)
Ventilator Days		0 (0)	0 (0)
Admission Acuity	ED only	2	2
	Floor	2	2
	Floor, then ICU	1	0
	ED to ICU	0	1
	Unknown	0	0
TEG Reaction Time (minutes)		5.4 (1.1)	7 (0.9)
TEG Maximum Amplitude (mm)		63.7 (7.5)	67.3 (0.8)
TEG Functional Fibrinogen Maximum Amplitude (mm)		20.5 (15.9)	26 (4.2)
TEG Lysis (%)		1.1 (1.5)	0.1 (0.1)
Aggregometry Baseline Impedance (resistance units)		1373 (50)	1395 (24)
Aggregometry ADP AUC (units)		58 (36)	69 (15)
Aggregometry Collagen AUC (units)		79 (45)	79 (26)
Aggrogometry Thrombin Analog AUC (units)		111 (34)	104 (26)
Aggregometry Arachidonic Acid AUC (units)		29 (25)	68 (48)
Aggregometry Ristocetin AUC (units)		59.5 (34.5)	108 (79)
PAI-1 (pg/mL)		2.6 (1.8)	4.5 (0.7)
Antiplasmin (% Activity)		96 (18.8)	93.5 (4.3)
Fibrinogen (mg/dL)		318 (114.2)	393 (129)
D-dimer (μg/dL)		1.4 (0.4)	0.8 (0.6)
Factor VIII (% Activity)		139.5 (81)	105.5 (107)
vWF (% Activity)		161 (34.5)	115 (50.7)
VEGF (pg/mL)		22.7 (14.8)	39.8 ()
Thrombomodulin (pg/mL)		5215 (2853)	4520 (970)
Plasminogen (% Activity)		78 (4.5)	101.5 (35.5)
Protein C (% Activity)		118.5 (24.4)	88.8 (25.2)
P-selectin (pg/mL)		21.8 (13.8)	32.9 (12.2)
tPA (pg/mL)		2823 (1075)	809.8 (375.2)
IL-8 (pg/mL)		8 (4.6)	10.9 (2.9)
TNFR1 (pg/mL)		1031.2 (669.2)	1499.3 (705.8)
Ang1 (pg/mL)		6114 (1348)	5718 (2958)
Ang2 (pg/mL)		833.2 (3419.2)	1270 (651.9)
MMP8 (pg/mL)		895.8 (555.7)	1759.8 (951.6)
SPD (pg/mL)		7717 (6317)	2664.7 (12015.3)
TREM1 (pg/mL)		301.8 (93.9)	119,9 (73.9)
IL-18 (pg/mL)		204.8 (34.47)	293.5 (143.1)
Cell-free Hgb (pg/mL)		203.4 (290.1)	63 (101.9)
IP10 (pg/mL)		22.4 (25.2)	429.1 (128.5)
IL-10 (pg/mL)		1.3 (1.3)	9.3 (1)
RAGE (pg/mL)		686 (45)	2476 (2349)

Continuous data are presented as median (interquartile range), except maximum O_2_ requirement, which is presented as median (minimum-maximum). BMI, body mass index; ICU, intensive care unit; ED, emergency department; TEG, thromboelastography; ADP, adenosine diphosphate; AUC, area under the curve; PAI-1, plasminogen activator inhibitor 1; vWF, von Willebrand Factor; VEGF, vascular endothelial growth factor; tPA, tissue plasminogen activator; IL-8, interleukin 8; TNFR1, tumor nerosis factor receptor 1; Ang1, angiopoietin 1; Ang2, angiopoietin 2; MMP8, matrix metalloprotease 8; SPD, surfactant protein D; TREM1, triggering receptor expressed on myeloid cells 1; IL-18, interleukin 18; Hgb, hemoglobin; IP10, interferon-gamma induced protein 10; IL-10, interleukin 10; RAGE, receptor for advanced glycation end products.

### 3.2 Plasma treated healthy platelets

**Figure 2:**
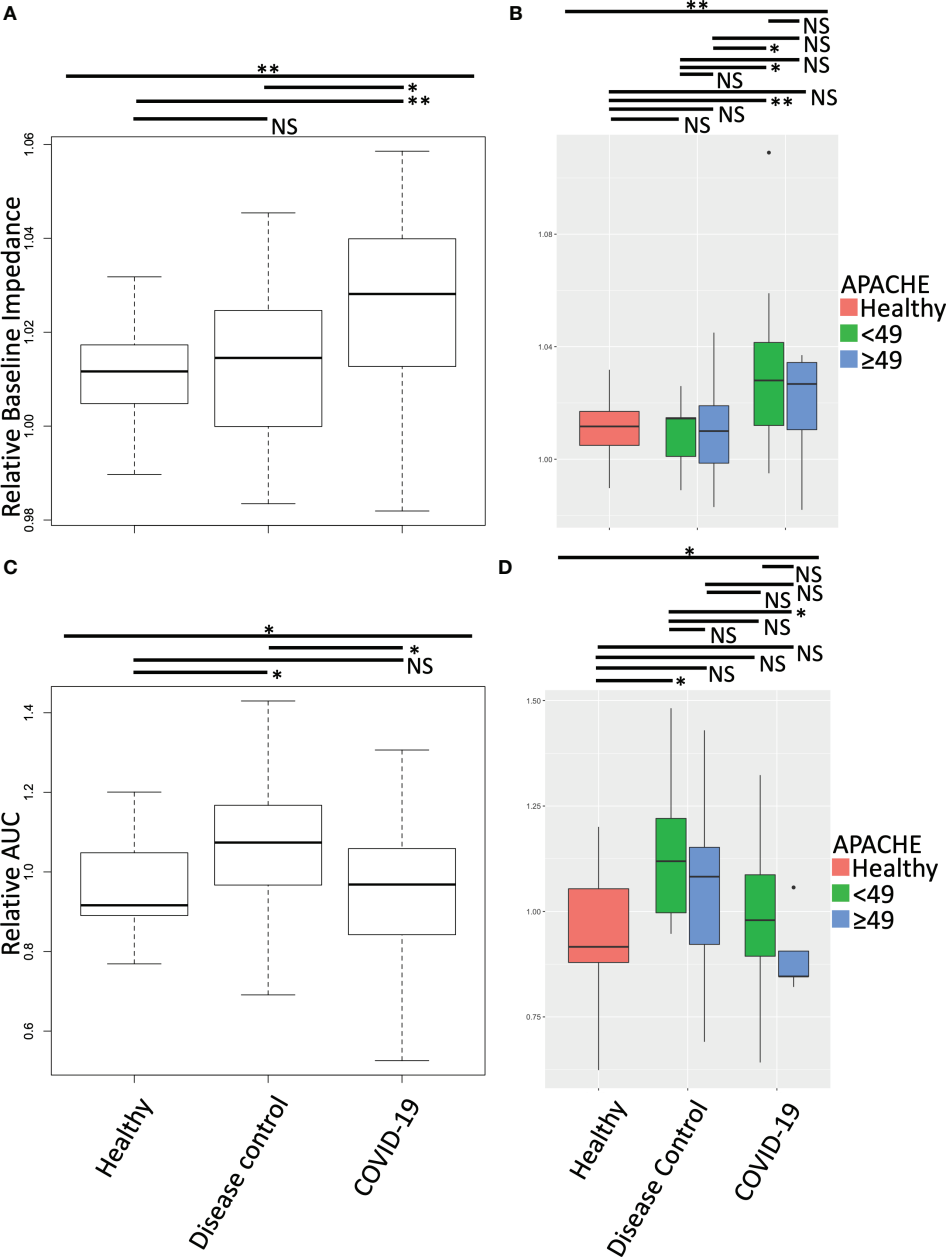
Aggregation effect of healthy platelets treated with plasma from COVID-19 patients and disease controls was measured by multiple electrode impedance aggregometry. Following treatment of isolated healthy donor platelets with plasma from COVID-19 patients and disease controls, multiple electrode impedance aggregometry was performed and **(A, B)** platelet aggregation prior to stimulation (baseline impedance), and **(C, D)** following collagen stimulation were measured. All data were normalized to autologous healthy donor plasma treatment performed in tandem, and compared to treatment with heterologous healthy donor plasma. **(A, C)** Treatment of healthy platelets with plasma from COVID-19 patients induced a statistically significant increase in platelet aggregation prior to stimulation (baseline impedance), but a decrease in response to collagen stimulation. Together these data suggest a platelet activating circulating milieu in COVID-19 that leads to a reduction in the responsiveness of platelets to further exogenous stimulation in *ex-vivo* assays. **(B, D)** To stratify patients, we performed a cut point analysis based on APACHE score of the plasma donors. The reduction in the response to exogenous stimulation was magnified following treatment with plasma from COVID-19 patients with higher compared to lower APACHE scores. Platelets from one of two male or two female healthy donors were treated with plasma from seven healthy donors, 12 disease control, and 14 COVID-19 patients. All plasma treatments were normalized to autologous healthy donor plasma treatment collected in tandem. Data were tested for normal distribution and statistical significance was assessed by one-way ANOVA followed by Tukey’s HSD *post hoc*. NS, not statistically significant; *, *p ≤* 0.05; **, *p ≤* 0.01.

To understand the impact of the circulating soluble environment of COVID-19 on platelet function we treated healthy platelets with plasma from COVID-19 patients and disease controls. Following plasma treatment of the platelets, we performed multiple electrode impedance aggregometry (**Figure 2**), measuring platelet aggregation response prior to stimulation (**Figures 2A, B**) and in response to collagen stimulation (**Figures 2C, D**). COVID-19 patient plasma treatment of healthy platelets increased their baseline impedance prior to stimulation with collagen (**Figure 2A**), and decreased their response to collagen stimulation compared to disease control plasma treatment (**Figure 2C**). This held true when plasma donor patients were clinically stratified by cut point analysis of degree of illness using the American Physiology and Chronic Health Evaluation (APACHE III) score (**Figures 2B, D**). Treatment of healthy platelets with plasma from COVID-19 patients with higher APACHE scores compared to lower APACHE scores induced similar increases in baseline impedance prior to stimulation with collagen, but a much larger decrease in response to stimulation with collagen (**Figure 2D**). Together these data suggest the presence of a platelet activating soluble milieu in COVID-19 that leads to a reduction in the circulating platelet response to exogenous stimulation in *ex-vivo* assays. This reduction in the response to exogenous stimulation was magnified when platelets were treated with plasma from COVID-19 patients who were sicker as measured by higher APACHE scores.

**Figure 3:**
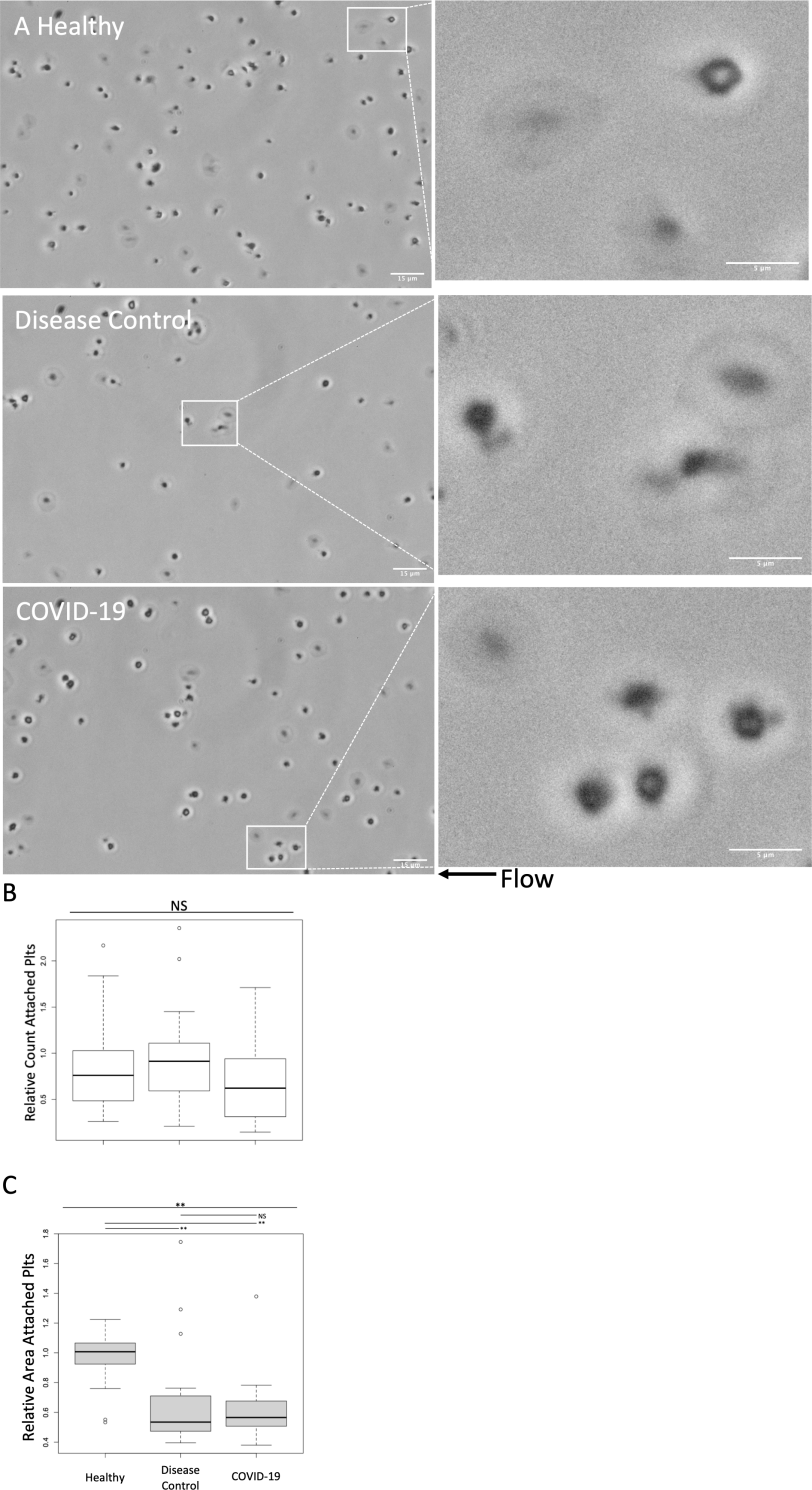
Adhesion and morphologic effects of healthy platelets treated with plasma from COVID-19 patients and disease controls was measured in a microfluidic adhesion assay. Healthy donor platelets were treated with plasma from COVID-19 patients, and healthy and disease controls, and then were passed through a collagen and thromboplastin coated chamber at 100s^-1^ (approximately venous shear). Unattached platelets were washed out at 1000s^-1^ (approximately arterial shear). Attached platelets were examined by phase contrast microscopy at 60X. **(A)** Representative images. In general, compared to healthy plasma treated platelets, treatment with plasma from both COVID-19 patients and disease controls induced platelets to adopt small and rounded phenotypes. Scale Bars are 15μm and 5μm for zoomed out and zoomed in panels, respectively **(B)** Attached platelets in five 60X fields were counted and counts were normalized to an autologous plasma treated group performed in tandem. The number of adhered platelets did not differ significantly between groups **(C)** Area of attached platelets was quantified to further examine morphological changes. Adhered platelets were significantly smaller following treatment with plasma from both COVID-19 patients and disease controls compared to healthy. There was not, however, a significant quantitative difference between treatment with plasma from COVID-19 patients compared to disease controls. Overall, the morphologic changes suggest that the circulating milieu in both COVID-19 patients and disease controls induced similar adhesive effects on healthy platelets. Treatment with plasma from COVID-19 patients did induce a qualitative increase in morphologic process development. Platelets from one of two male or two female healthy donors were treated with plasma from seven healthy donors, 12 disease control, and 14 COVID-19 patients. All plasma treatments were normalized to autologous healthy donor plasma treatment collected in tandem. Statistical significance was assessed using the Kruskal-Wallis test followed by pairwise Wilcoxon Signed-Rank tests. NS, not statistically significant; *, *p ≤* 0.05; **, *p ≤* 0.01.

Plasma treated healthy platelets were also subjected to adhesion assays in a collagen and thromboplastin coated parallel plate microfluidic flow chamber (**Figure 3**). There was no significant difference in adhesion of healthy platelets treated with plasma from COVID-19 patients, disease controls, and healthy controls (**Figure 3B**). While the number of adhered platelets did not differ, the morphology of the adhered platelets differed (**Figure 3A**). Compared to healthy plasma treated platelets, treatment with plasma from both COVID-19 patients and disease controls induced platelets to adopt small and rounded phenotypes. As a means of quantifying the observed morphologic changes, we measured the area of adhered platelets (**Figure 3C**). Adhered platelets were significantly smaller following treatment with plasma from both COVID-19 patients and disease controls compared to healthy controls. Although there was not a significant quantitative difference in the measured area of adhered platelets between those treated with plasma from COVID-19 patients compared to disease controls, treatment with plasma from COVID-19 patients qualitatively induced increased process development compared to disease controls.

Overall, the circulating milieu in both COVID-19 patients and disease controls induced similar aggregatory and adhesive effects on healthy platelets, but treatment with plasma from COVID-19 patients induced a reduction in the platelet aggregation response to exogenous stimulation compared to disease controls, and a qualitative increase in morphologic process development.

### 3.3 Platelet releasate treated healthy neutrophils

**Figure 4:**
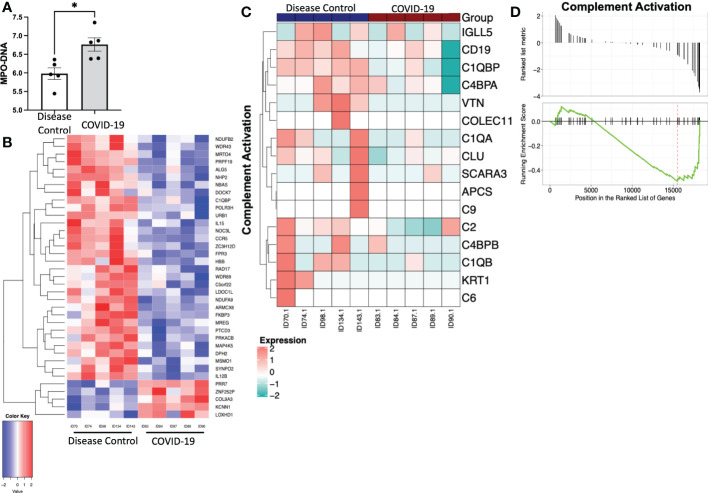
Treatment of healthy neutrophils with platelet releasate from COVID-19 patients and disease controls. **(A)** Myeloperoxidase-deoxy ribonucleic acid (MPO-DNA) complexes were quantified from COVID-19 patient and disease control platelet releasate treated neutrophils, measured with a capture ELISA. Data presented as optical density standardized to negative control. *, p<0.05.**(B)** Heatmap with hierarchical clustering displaying transcriptomic changes between treatment with platelet releasate from COVID-19 patients and disease controls. Bulk RNA sequencing results of gene changes requiring a fold change >2 and p-value <.05. There are 5 upregulated genes and 32 downregulated genes. N = 5. **(C)** Heatmap and **(D)** plot displaying the gene set enrichment pathway analysis of complement activation (Enrichment score=-0.493, p=0.038). N = 5.

To determine if the contents released from platelets in COVID-19 patients induce a proinflammatory neutrophil phenotype, we isolated platelets from patients with COVID-19 and disease controls and collected releasate from those platelets following stimulation with CaCl_2_. We then treated healthy neutrophils with these platelet releasates. Treatment with platelet releasate from COVID-19 patients caused healthy neutrophils to produce more MPO-DNA complexes (**Figure 4A**). Additionally, treatment with platelet releasate from COVID-19 patients caused healthy neutrophils to have distinct differentially expressed genes when compared to those treated with platelet releasate from disease controls (**Figure 4B**). Genes in the complement pathway were significantly downregulated in healthy neutrophils treated with platelet releasate from COVID-19 patients compared to disease controls (*p*<0.05) (**Figures 4C, D**). Overall, this suggests that immunomodulatory paracrine factors are released from platelets in COVID-19 patients distinct from disease controls.

## 4 Discussion

COVID-19 is associated with prothrombotic proinflammatory biologic profiles thought to mediate the thromboinflammatory clinical complications. Although evidence has accumulated that platelets and neutrophils are both crucial cellular contributors to the thromboinflammation of COVID-19 (25), therapeutic targeting of these cellular contributors to improve morbidity and mortality of COVID-19 has proven difficult (26). Further, the effect of the circulating soluble environment on this platelet-neutrophil axis has not been fully elucidated. As such, we tested the hypotheses that 1) plasma from patients with COVID-19 can induce a prothrombotic platelet functional phenotype, and that 2) contents released from platelets (platelet releasate) from COVID-19 patients can induce a proinflammatory neutrophil phenotype. To explore this, we prospectively collected early plasma samples from COVID-19 patients (before receipt of any therapeutics) and in parallel collected plasma samples from symptom-matched COVID-19 negative disease controls to assess the effects of COVID-19 plasma on healthy platelet aggregation, adhesion, and morphology. In addition, we created platelet releasates from platelets isolated from COVID-19 patients and disease controls to assess the indirect non-contact mediated effects of COVID-19 platelets on healthy neutrophil NET-formation and transcriptomic profiles.

First, we found that when healthy platelets were exposed to these plasma environments, the COVID-19 patient plasma induced platelets to have an auto-aggregatory profile and altered adhesion characteristics similar to treatment with disease control plasma, with one notable difference. COVID-19 patient plasma induced a functional phenotype consistent with what has been described as a ‘functional exhaustion’ in other disease states (27–29). This was evidenced by the induction of significantly increased platelet aggregation in response to the plasma treatment alone prior to exogenous stimulation with agonists and increased morphologic process development, yet a weaker aggregation response following collagen stimulation. These *ex-vivo* experimental observations suggest that the circulating soluble thromboinflammatory milieu of COVID-19 elicits a stronger functional effect on platelets than that of other disease, capable of reducing or exhausting the ability of platelets to aggregate further in response to additional surface stimulation. This biology could be hypothesized to contribute to an overall lack of success in decreasing microthrombosis associated complications in COVID-19 clinical trials with anti-platelet therapies (30–32). If the circulating platelets are in an environment that causes a ‘functional exhaustion’, it may be that further platelet inhibition may not be beneficial.

Second, platelets themselves contribute to the soluble circulating environment as they release a rich repertoire of contents that are capable of noncellular and cellular control of coagulation and inflammation (5, 7). As such, we explored the contribution of platelets to the soluble environment by creating platelet releasates from platelets isolated from COVID-19 patients and disease controls and treating neutrophils with these releasates to assess the paracrine platelet-neutrophil axis. Treating healthy neutrophils with platelet releasate from COVID-19 patients induced evidence of NETosis and differential regulation of complement pathway genes. These findings were pronounced in comparison to the treatment of healthy neutrophils with platelet releasate from disease controls.

NETs are known pro-thrombotic scaffolds (33). Previous work has suggested that NETosis in COVID-19 is due to binding of SARS-CoV-2 to neutrophil angiotensin converting enzyme 2 (15, 20), and also due to interactions with platelets (7). NETs have been found in occlusions of micro-vessels in COVID-19 (34). Further, platelet factor-4 has been found to be elevated in COVID-19 patient plasma, and evidence of NETosis was found in COVID-19 patient lung tissue that was also rich in platelet factor-4 (7). Platelets, themselves, have been shown to be hyperreactive in COVID-19 patients (4, 35). There has been some suggestion that this could potentially result from direct contact between SARS-CoV-2 and platelets (36). However, evidence for SARS-CoV-2 internalization in platelets is scant (4, 37). The actual mechanism more likely involves multiple inputs. Classically, neutrophils (and other leukocytes) are viewed as being recruited to sites of inflammation or endothelial damage by platelets through a combination of chemoattractant and direct cell-cell interaction, to allow them to extravasate and carry out immune-inflammatory functions (38). *In-vivo*, this is likely to be highly complex and include a process of paracrine signaling from platelets to leukocytes (39, 40). Overall, our observations expand on these existing findings by demonstrating that the soluble circulating environment of COVID-19 may also have important effects on the described adhesion and aggregation behavior of platelets, and further that those platelets are critical effector cells of neutrophils *via* a cell-cell contact independent paracrine line of communication in COVID-19 patients. Together our data further highlight a link between thrombotic and inflammatory mediators in COVID-19 that may be more pronounced and/or unique compared to other diseases. With this insight, it is possible that platelets’ contribution to thrombosis in COVID-19 could leverage the inflammatory side of the thromboinflammatory continuum. As previously mentioned, platelet inhibition has not been a panacea in the context of COVID-19. However, platelets’ role as an inflammatory paracrine effector cell may represent a therapeutic avenue.

There are limitations to these studies. Regarding the *ex-vivo* assays, the sample size of platelet releasate treated neutrophils is low. A single neutrophil donor was chosen to limit donor heterogeneity in sequencing experiments, as the primary goal was to compare the effects of platelet releasate from COVID-19 patients and disease controls on neutrophils, thus the differences observed should only be due to treatment. However, the disease control releasates were skewed towards male. Regarding the plasma treated platelets, measuring baseline impedance is a proxy for auto-aggregation, but we do not have confirmatory surface marker expression or other measures of their activation or aggregation status. Finally, regarding our plasma and platelet releasate donors, it should be noted that although the data demonstrate evidence of a generally prothrombotic proinflammatory phenotype of the COVID-19 patients relative to the disease controls, the study was not powered to detect differences in these biomarkers, and the differences were small overall. This may be important given that much of the COVID-19 literature lacks parallel prospectively collected disease control samples for comparison, and either rely on previously collected specimens for disease controls or use healthy control comparisons alone. As such, these methods may risk overestimation of the procoagulant and proinflammatory phenotypes of COVID-19. Importantly, all samples included were collected on emergency department presentation for both COVID-19 patients and disease controls, which is novel compared to much of the COVID-19 literature in which samples were collected in admitted patients later in their disease courses and often after initiation of anti-viral and anti-inflammatory therapies, another aspect that could contribute to the small differences overall from the disease controls.

Finally, it is not clear what is in the plasma and released from the platelets that is responsible for the cell-cell contact independent effector cell biology that we identified. Unfortunately, we were unable to perform characterization studies of the platelet releasate samples described here. Platelet releasates were prepared in very limited volume because they were made from whole blood obtained under an initial waiver of consent, which was central to obtaining these samples prior to any therapeutics in sick patients. As such, we were able to prepare only very limited quantities that were used in the experiments. Of note, among the biomarkers we measured in the plasma, PAI-1 was different between COVID-19 patients and disease control donors. PAI-1 has anti-fibrinolytic function, is differentially regulated by diverse cytokines including those involved in inflammation (41). PAI-1 is a driver of hypofibrinolysis in COVID-19 (42). Interestingly, PAI-1 has been suggested to regulate and be regulated by inflammatory biomarkers, namely IL-6, thus linking thrombotic and inflammatory processes (43, 44). A large reservoir of PAI-1 resides in platelets themselves, and active PAI-1 is displayed on the platelet membrane on activation (45–47). Exposure to PAI-1 has been shown to have pleiotropic effects on neutrophils, including promotion of tissue infiltration and ischemia reperfusion injury (48–50). This may be relevant to our findings that platelet releasate from patients with COVID-19 caused healthy neutrophils to increase NET formation and alter their transcriptional program related to complement. Testing the involvement of PAI-1 in our observations is warranted. In addition, several biomarkers of inflammation were upregulated in COVID-19 patients compared to the disease controls donors, namely IP-10, IL-10, and RAGE. IP-10 is best known for its role as a leukocyte chemoattractant and inhibitor of endothelial cell proliferation (51). Although the canonical role of IL-10 is as an anti-inflammatory cytokine, there has been some evidence that in COVID-19 it may exert a pro-inflammatory effect (52). RAGE is expressed in alveolar epithelium and is associated with lung injury, acute respiratory distress syndrome, and, more recently, COVID-19 severity (53–55). Importantly, the biomarker profiles of the plasma used to treat platelets were similar to those in the plasma of patients whose platelets were used to generate platelet releasate samples.

In summary, our work suggests that in COVID-19, platelets are exposed to a procoagulant proinflammatory environment that leads to auto-aggregation, morphologic change, and may induce a ‘functional exhaustion’. Further, our data supports that the platelets themselves may contribute to the soluble circulating milieu and *via* cell-cell contact independent paracrine effects can alter neutrophils at both the transcriptional and functional levels. Other investigators have noted PAI-1 as well as vWF decorating NETs in the setting of ischemic stroke (56), and NETs have a well know role in thrombus formation (57). Given our observations of increased plasma levels of PAI-1 in COVID-19 patients, and increased NET formation following treatment of healthy neutrophils with platelet releasate from COVID-19 patients, there is potential that platelets may also play a cell-cell contact independent paracrine role in COVID-19 hypercoagulability *via* signaling to neutrophils.

## Data availability statement

The original contributions presented in the study are publicly available. This data can be found here: Gene Expression Omnibus (GEO) with accession ID: GSE225217.

## Ethics statement

The studies involving human participants were reviewed and approved by Institutional Review Board/Human Research Protection Program of the University of California, San Francisco. The patients/participants provided their written informed consent to participate in this study.

## Author contributions

All authors participated in the writing and editing of the manuscript. AF assisted in study design, designed experiments, performed experiments, analyzed data, and prepared the manuscript. EA designed experiments, performed experiments, and analyzed data. CK designed experiments, performed experiments, and analyzed data. ZM assisted in study design, designed experiments, performed experiments analyzed data. KH performed experiments. BN-G managed clinical sample and data collection. CH assisted in study design. RB assisted in study design, designed experiments, performed experiments, analyzed data. CJ performed experiments. KW performed experiments and analyzed data. SL performed experiments, and analyzed data. J-HL performed experiments, and analyzed data. Y-PY performed experiments, and analyzed data. MM assisted in study design, and designed experiments. TB assisted in study design. JB assisted in study design. MN assisted in study design, and designed experiments. LK assisted in study design, designed experiments, analyzed data, and prepared the manuscript. All authors contributed to the article and approved the submitted version.

## Additional Co-ACIT Study Group members

Biniam Ambachew^1^, Sarah Cary^4^, Lauren Chalwell^9^, Christopher Colwell^9^, Clayton Josephy^3^, Deanna Lee^1^, Matthieu LeGrand^4^, Juan Carlos Montoy^9^, Aaron E. Kornblith^9^, Philip Kurien^4^, Brenda Nunez-Garcia^1^, Viet Nguyen^1^, John J. Park^1^, Arun Prakash^4^, Brittany Robinson^1^, India Shelley^1^.

^1^ Department of Surgery, University of California, San Francisco; Zuckerberg San Francisco General Hospital; San Francisco, CA, USA

^2^ Department of Surgery, University of Pittsburgh; Pittsburgh, PA, USA

^3^ Division of Pulmonary, Critical Care, Allergy and Sleep Medicine, Department of Medicine, University of California, San Francisco; San Francisco, CA, USA

^4^ Department of Anesthesia and Perioperative Care, School of Medicine, University of California, San Francisco; San Francisco, CA, USA

^5^ Cardiovascular Research Institute, University of California, San Francisco; San Francisco, CA, USA

^6^ Leon H. Charney Division of Cardiology, Department of Medicine, NYU Grossman School of Medicine; New York, NY, USA

^7^ NYU Center for the Prevention of Cardiovascular Disease, NYU Langone Health; New York, NY, USA

^8^ Division of Vascular Surgery, Department of Surgery, NYU Grossman School of Medicine; New York, NY, USA

^9^ Department of Emergency Medicine, University of California, San Francisco; San Francisco, CA, USA
